# Effects of Different Ratios of Sewage Sludge and Cattle Manure on Growth and Propagation of *Eisenia Fetida*

**DOI:** 10.1371/journal.pone.0156492

**Published:** 2016-06-03

**Authors:** Yukui Li, Qingchuan Liu, Fei Liu, Pengfei Zhu, Lichao Zhang, Xiujie Zhou, Chongyu Sun, Yunhuan Cheng

**Affiliations:** 1 School of Environmental Sciences, Huaibei Normal University, Huaibei, Anhui, China; 2 College of Food Science, Hefei University of Technology, Hefei, Anhui, China; University of Delhi, INDIA

## Abstract

Domestic sewage sludge and cattle manure are rich in nutrition elements, but without proper disposal, are harmful to the environment. Here with an indoor culture method, we used *Eisenia fetida* to dispose different ratios of sewage sludge and cattle manure, and thereby investigated the effects and acting rules of these sludge-manure mixtures on the growth and reproduction of *E*. *fetida*. We find these mixtures are food sources for *E*. *fetida*, and their physiochemical properties are significantly changed after disposal by earthworms. Paired samples t-test shows the average change after different treatments is -20.37% for total organic carbon, 85.71% for total Kjeldahl N, -6.67% for total P, 8.33% for pH, -24.78% for EC (ms·cm^-1^), and -57.10% for C/N ratio. The average growth rate after treatment CD-70 is 9.20 mg·worm^-1^·day^-1^; the average growth rates of *E*. *fetida* on day 0–28, day 29–56, and day 57–91 are 9.33, 11.90 and 6.95 mg·worm^-1^·day^-1^, respectively, indicating a trend of "rapid—rapidest—slow" growth. Other treatments all show this trend. Though all earthworms developed reproductive rings during the test periods, the appearing time and the cocoon production time both differed among these treatments. The cocoon production amount is maximized to 233 after treatment CD-70. The cocoon production rates are significantly different among these treatments, and the maximum and mean are 0.32 and 0.17–0.32, cocoons·worm^-1^· day^-1^, respectively. *E*. *fetida* can modestly enrich Cd, but is not very effective over Sb or other heavy metals. *E*. *fetida* can remove a part of heavy metals from sewage sludge and cattle manure. Generally, the mixtures of sewage sludge and cattle manure can largely affect the growth and propagation of *E*. *fetida* in a ratio-dependent way.

## Introduction

As is well-known, a huge amount of organic solid wastes, including urban sludge, crop straw, cattle manure, sheep manure and pig manure, is produced from planting, breeding, and industrial fields. These wastes, without recycled use, will deteriorate the environment, so their disposal and management become urgent [1–2]. Like other developing countries, China is challenged by the inappropriate and arbitrary disposal of solid wastes. The common disposal methods, including burning and land use, cause severe pollution to air, soil and groundwater. On the contrary, earthworm composting is an environmental-friendly and sustainable technique, as it can efficiently decompose organic waste, save energy resources and avoid secondary pollution [3–4].

Earthworms have been successfully applied into disposal of industrial, agricultural and urban wastes, and the main species include *Eisenia foetida*, *E*. *andrei*, *E*. *eugeniae* and *Perionyx excavatus* [5]. In particular, *E*. *fetida* is widely used in earthworm composting because it is easy-to-raise. Its biological properties are extensively reported. *E*. *fetida* can decompose organic waste, excrete the so-called wormcast, and synthesize earthworm biomass [6].

As reported, earthworm composting is a low-cost, useful and competitive technique for the disposal of sewage from sewage treatment plants [7]. Wormcast resulting from earthworm treatment is a high-quality organic fertilizer and has very high economic and environmental values. The nutrients in wormcast, such as N, P and K, can be more easily absorbed by plants.

*E*. *fetida* can use different solid wastes as foods, including legume litter [8], sewage sludge, activated sludge, rabbit manure, cattle manure, pig manure, and sheep manure. So far, there are many studies and applications about earthworm-based disposal of agriculture wastes, including cattle manure which provides a more nutritious environment for earthworm composting. As reported, when *E*. *fetida* was fed with mixtures containing different ratios of sugarcane bagasse to cattle manure, the growth and reproduction of *E*. *fetida* were both maximized at the ratio of 50:50, but were both significantly affected by higher concentrations of bagasse waste [9]. In order to evaluate the potential genotoxicity of pressmud to onion, Bhat et al. [10] prepared composting mixtures with different ratios of pressmud to cattle manure and found this technique helped to reduce the genotoxicity of pressmud. According to Xie et al. [11] the 60-day experiment of vermicomposting shows that 100% sludge is suitable for the growth and fecundity of earthworms, while the addition of CD into sludge significantly improved the worm biomass and reproduction, as it improved the environment for earthworm fecundity.

The biomass growth rate and cocoon production of earthworms feeding on cattle manure are both higher compared with the application of sheep manure [12–14]. However, there is little research about how *E*. *fetida* disposes sludge and cattle manure mixtures.

The objective of the present study is to thoroughly uncover how mixtures of sewage sludge and cattle manure would affect the growth and propagation of *E*. *fetida* (Oligochaeta), and thereby to underlie the earthworm-based processing of solid wastes. We would investigate: (i) how the ratios of sewage sludge to cattle manure could affect the growing rate, sexual development and daily cocoon production rate of *E*. *fetida*; (ii) the rules underlying how the sludge-to-manure ratios affect the physiochemical properties after earthworm processing; (iii) to ascertain the changes of the concentration of heavy metal of mixtures in the different treatments.

## Materials and Methods

### Ethics Statement

All animal experiment procedures were approved by the Institutional Research Ethics Committee of Huaibei Normal University. Samples (sludge) were collected from a local farm of private land, and permitted by the owner. Our sampling (cattle manure) for research was approved by the Institutional Review Board of Huaibei Sewage Treatment Plant, a large scale enterprise.

### Materials

Cuboid plastic buckets (35×25×18 cm^3^) used here had permeable holes in the bottom and installed with gauze nets at the bottom, preventing the escape of *E*. *fetida*. Each bucket was placed with 2000 g (dry weight) of materials and 8 physically-healthy earthworms with similar sizes. Five treatments were set, with each tested in sextuplicate. After inoculation, the bucket mouths were covered with fine gauze, which prevented *E*. *fetida* from escape. The buckets were irrigated once every 1–2 days.

Test earthworms: *E*.*fetida* was purchased from a local factory, Huaibei Runhua Biotech Co. Ltd. Before tests, the soils on the earthworms were washed away by deionized water, and then the water was sucked off. In each test, each earthworm was weighed to be about 120 mg. The total biomass was about 1.0 g. The earthworms (about 20 days old and immature) were placed in an artificial climate box (20 to 25°C) for about 3 days of pre-culture.

Test sewage sludge and cattle manure: The sludge was collected from Huaibei Sewage Treatment Plant in November 2013. In this plant, a Carrousel oxidation ditch was used in sewage disposal. Before treatment in the oxidation ditch, the grit chamber was already precipitated with abundant inorganic matter and insoluble sand. The sludge in the final settling tank was flocculents mainly composed of aerobic bacteria, as well as protozoa attached to colloidal substances. The sewage sludge after dehydration contained about 75% moisture. The cattle manure was collected from a farm in November 2013. This place is a mining-caused subsided land and has been levelled to Xiqiang Dairy Cow Breeding Base (N 33°56′39″, E 116°49′15″). The physiochemical properties of the sewage sludge and cattle manure are listed in Table 1.

**Table 1:** Physicochemical characteristics of tested composting material (mean ± S.D).

Parameters	Cattle manure (g)	Sewage sludge (g)
**CD-0**	0	2000
**CD-30**	600	1400
**CD-50**	1000	1000
**CD-70**	1400	600
**CD-100**	2000	0
**TOC (%)**	42.7±1.28	34±1.54
**TKN (%)**	1.26±0.15	2.34±0.21
**TP (%)**	1.35±0.11	1.26±0.07
**pH**	7.2±0.02	7.8±0.03
**EC (ms·cm**^**-1**^**)**	3.43±0.05	2.58±0.09
**C/N ratio**	33.8±1.06	14.5±1.37

Five mixtures with different ratios of sludge to cow manure were prepared and used as substrates in the 91-day experiments. The weights and cocoon yields of earthworms were measured weekly, while the sexual development stage was checked every week. Biomass and cocoon production rate of each earthworm were measured as described by Singly and Suthar [15].

To improve the disposal efficiency, we had to correctly determine the optimal ecological conditions during *E*. *fetida* decomposition of urban sludge. During earthworm disposal of sewage sludge and cattle manure, the moisture content and temperature both had great effect on the growth and reproduction of *E*. *fetida*. Regarding these facts, we set the test conditions as follows: moisture content 65% in sewage sludge and cattle manure, temperature at about 25°C, pH 7.2, and C/N ratio = 33.8.

### Chemical analysis

Double distilled water (water soil ratio = 5: 1) was added to the wind-dried substrates, which had passed a 1 mm screen. After 20 minute of oscillation, the materials were filtered through Whatmann No.1 membranes, and the electroconductibility (EC) and pH in the tested solutions were determined. Total organic carbon (TOC) was measured as follows: 0.50 g of a dry sample, in a crucible (weight known), was oven-dried at 105°C for 12 h; then the crucible was immediately taken out, put into a dryer and after cooling, was weighed. Then the crucible was transferred into a muffle, which was heated to 550°C and burnt for 1 h; then the crucible was removed to the dryer and after cooling, was weighed [16]. Total P (TP) was measured via the perchloric acid—sulfuric acid boiled Mo Sb colorimetric method. Specifically, to a crucible, a dried sample (0.25 g, after passing 100-mesh sieve) was put inside, added with 3–4 drops of absolute ethanol, and then was spread with 2 g of NaOH. Then the crucible was put into a high-temperature furnace, which was heated via a gradient to 720°C. Then the cucible was taken out, cooled and added with water to constant volume for developing. Then the absorbance at 700 nm was detected so as to determine the P content [17]. Total Kjeldahl nitrogen (TKN) was measured via a modified international criterion. Specifically, to a digestion tank, 0.50 g of a dried sample was put inside, added with mixed acid, heated on an electric hot plate at low temperature for 0.5 h, and then taken out and cooled down. A mixed catalyst (1.40 g) was added, through a dry long-neck funnel, into the bottom of the digestion tank, which was heated on the furnace. The digestion temperature was determined to be when the solution slightly boiled and the sulfuric acid condensate was about 1/3 from the bottom of the digestion tank. The sample was digested until it became grey green. Then after the solution become transparent, it was digested for at least 2 h, until the ammonium salt reacted completely. After digestion, the digestion tank was taken out, cooled down, and connected to a Kjeldahl apparatus for distillation. To each sample distillate after complete pretreatment, 2–3 drops of a bromocresol green-methyl red mixed indicator were added. The resulting solution was titrated with the standard HCl solution until the color changed from blue-green to permanent light pink. The total used volume of HCl solution (0.12N) was recorded and used to compute TKN [18]. Total content of heavy metals including Sb was detected as follows: 0.10 g of a sample (passing 100-mesh sieve) together with 6 mL of aqua regia and 2 mL of HF was microwave-digested [19]. After that, the solution was added with 1–2 drops of perchloric acid, and then acid-removed on an electric hot plate at 150°C for 2 h. After addition with water to the mark, and filtration, the solution was stored in a refrigerator at 4°C, and detected via inductively coupled plasma emission spectrometry.

### Computation and data analysis

Relevant indices were measured and computed as follows:

$$\text{Growth rate }=\frac{\text{Total earthworm weight after test - initial earthworm weight}}{\text{Initial earthworm weight }\times \text{ breeding time}}$$ (1)

$$\text{Daily cocoon production rate }=\;\frac{\text{Cocoon production amount}}{\text{Number of earthworms }\times \text{ test period}}$$ (2)

$$\text{C/N ratio }=\frac{\text{TOC}}{\text{TKN}}$$ (3)

All data are expressed as mean ± standard deviation and analyzed on SPSS 17.0. The physiochemical properties before and after disposal were compared by paired t-test, with significance level set at 95% reliability. The growth rate and daily cocoon production rate of *E*. *fetida* were tested via one-factor analysis of variance (ANOVA). The differences among five treatments were compared by least significant difference (LSD) multiple test at the confidence interval of 95%.

## Results and Discussion

### Changes in physiochemical properties of mixtures

The physiochemical properties of materials after earthworm composting are listed in Table 2. Paired-samples T-test shows the TOCs are significantly changed after treatment CD-0 (t = 10.17), CD-30 (t = 15.65), CD-50 (t = 15.78), CD-70 (t = 62.50), CD-100 (t = 25.85) (all *P*<0.001), and especially after CD-70, the reduction degree is maximized to 33.65%.

**Table 2:** Changes of physicochemical quality of mixture under different ratios of sewage sludge and cattle manure.

Physicochemical characteristics	CD-0	CD-30	CD-50	CD-70	CD-100
TOC (%)					
Initial	34±1.39	36.61±1.51	38.35±2.32	40.09±1.78	42.7±1.55
Final	27.6±2.27	24.8±1.16	27.5±1.32	26.6±1.50	33.5±2.26
*t*-value	10.18***	15.65***	15.78***	62.50***	25.8***5
Percent change	-18.82	-32.26	-28.29	-33.65	-21.55
TKN (%)					
Initial	2.34±0.17	2.02±0.25	1.83±0.21	1.58±0.18	1.26±0.20
Final	2.65±0.13	2.34±0.21	2.16±0.22	1.77±0.19	1.35±0.22
*t*-value	-10.74***	-14.03***	-15.39***	-9.13***	-3.85*
Percent change	13.25	15.84	18.03	12.03	7.14
TP (%)					
Initial	1.26±0.14	1.28±0.17	1.31±0.16	1.33±0.11	1.35±0.13
Final	1.21±0.15	1.25±0.16	1.26±0.15	1.26±0.13	1.27±0.12
t-value	4.56**	2.09	5.0**	3.69*	5.09**
Percent change	-3.97	-2.34	-3.82	-3.82	-5.93
*p*H					
Initial	7.8±0.09	7.7±0.15	7.5±0.09	7.5±0.15	7.2±0.14
Final	6.6±0.17	6.9±0.09	6.7±0.13	6.8±0.09	6.4±0.09
*t*-value	14.01***	21.91***	30.98***	19.17***	11.71***
Percent change	-15.38	-10.39	-9.46	-9.33	-11.11
EC(ms·cm^-1^)					
Initial	2.58±0.09	2.74±0.07	3.16±0.10	3.25±0.08	3.43±0.12
Final	2.13±0.12	2.05±0.09	2.65±0.12	2.38±0.16	3.07±0.12
*t*-value	28.65	18.10	44.17	24.64	17.29
Percent change	-17.44***	-25.18***	-16.14***	-26.77***	-10.50***
C/N ratio					
Initial	14.58±0.97	18.3±2.18	21.1±1.65	25.5±1.76	34.4±4.2
Final	10.42±0.88	10.65±0.86	12.79±0.75	15.1±0.85	25.15±2.68
*t*-value	13.35***	11.85***	20.09***	22.99***	9.13***
Percent change	-28.32	-41.52	-39.25	-40.77	-26.78

Note: CD indicates cattle manure; C/N ratio indicates C to N ratio.

*: 0.01<*P*<0.05,

**: 0.001<*P*<0.01,

***: *P*<0.001 (the same below).

The mineralization of organic matter, which was released as CO_2_ into the air, was accelerated by earthworms, thereby reducing the organic content and improving the stability of wastes. Among all of the chemical parameters, TOC imposed the most significant effect on the growth and development of earthworms, because it was involved in the earthworm assimilation. Earthworms accelerate the organic decomposition owing to the synergy between earthworms and microorganisms. The microflora in front of and behind the digestive tracts both changed significantly. The addition of earthworms promoted the organic decomposition. The loss of organic carbon during earthworm composting is attributed to the digestion of the primary substrate carbohydrates and other polysaccharides [20]. The digestion by earthworms accelerated the mineralization and humification of organic matter. Moreover, a part of organic carbon was absorbed by earthworms to form their own body components, which were used in their growth and propagation.

Paired-samples T-test shows TKNs are significantly different after treatment CD-0 (t = -10.7, *P*<0.01), CD-30 (t = -14, *P*<0.01), CD-50 (t = -15.4, *P*<0.01), CD-70 (t = -9.12, *P*<0.01), and CD-100 (t = -3.85, *P*<0.05). TKN increases by 18.03% after treatment CD-50 and by 7.14% after treatment CD-100. TKN increases because organic wastes, the products from mineralization and earthworm assimilation, were digested by earthworms, while the nitrogen was concentrated in the wormcast [21]. As reported, the TKN of sugar mill effluent increases because nitrogen mineralization is accelerated by earthworm-induced waste decomposition [22]. The nitrogen surplus by microorganism intestine and earthworms stabilizes nitrogen excreta, mucus, enzyme and certain hormones, owing to the mineralization of organic matter during vermicomposting [23].

Paired-samples T-test shows TPs are significantly changed after treatment CD-0 (t = 4.56, *P*<0.01), CD-50 (t = 5.0, *P*< 0.05), CD-70 (t = 3.69, *P*< 0.05), and CD-100 (t = 5.09, *P*<0.05), but not after CD-30 (t = 2.09, *P*>0.05). The reason is that the in-vitro nitrification of ammonium salt and the in-vivo phosphate solublization by worm gut enzyme result in the increase of TP in vermicomposts [24]. Lee studied the organic content in gut where organic matter is soluble and stabilizes phosphorous [25].

Paired-samples T test shows pH significantly changes after treatment CD-0 (t = 14.01), CD-30 (t = 21.91), CD-50 (t = 30.99), CD-70 (t = 19.17), and CD-100 (t = 11.71) (all *P*< 0.01). The pH drops by -15.38% after treatment CD-0 and minimizes to 6.4±0.01 after treatment CD-100. The decline in pH during vermicomposting is due to the mineralization of N and P compounds, and the earthworm-related gut microorganisms contribute to the generation of humic and fulvic acids [26].

All earthworms died at pH < 5 or > 8, so the optimal pH for earthworm treatment of activated sludge is pH 5–8. The change of pH may be attributed to the production of fulvic acid and humic acid. It is believed that pH is likely to become neutral during earthworm composting [27–29].

The range of EC in the materials is 2.58–3.43 ms·cm^-1^ before treatments and 2.05–3.07 ms·cm^-1^ after treatments. Paired-samples T test shows ECs are significantly different after treatment CD-0 (t = 28.65), CD-30 (t = 18.10), CD-50 (t = 44.17), CD-70 (t = 24.64), and CD-100 (t = 17.30) (all *P*< 0.01). EC and pH are limiting factors on the growth and development of *E*. *fetida* [30]. The reduction of ECs is attributed to the loss of organic carbon and the release of different mineral salts. EC dropped by 26.77% after treatment CD-70, which is similar to a previous finding of 28.69% [31]. However, Karmegam and Daniel reported an increase in EC during vermicomposting, which is due to the increase of soluble salt level resulting from the mineralization by worms and the microbes existing in the earthworm gut and the organic matter [32].

Paired-samples T test shows C/N ratios are significantly changed after treatment CD-0 (t = 13.36), CD-30 (t = 11.85), CD-50 (t = 20.09), CD-70 (t = 22.99), or CD-100 (t = 9.13) (all *P*< 0.01). The C/N ratio is an important factor reflecting the growth and propagation of composting earthworms. The C/N ratio changes at the maximum rate of -41.52% after treatment CD-30. The causes are the reduction of organic carbon and the increase of total nitrogen, which is consistent with other studies. In nearly all experiments, when earthworms were observed, the C/N ratio significantly dropped with time. As reported, the C/N ratios in the resulting materials of crop residue and cattle manure mixtures were smaller after the addition of earthworms compared with the case of no earthworms, indicating earthworms can accelerate the reduction of C/N ratio [33–35].

Earthworms accelerated the mineralization of organic matter, which was released as CO_2_ into the air, thereby reducing the organic content. The loss rate of organic nitrogen during ammoniation is far smaller than that of organic carbon, which led to the gradual reduction of C/N ratio until stabilization. Under normal circumstances, the optimal C/N ratio is 25–35. At C/N ratio = 25, the reproductive rate and feeding capacity were both maximized, but the wormcast had very high fertility and smallest environmental pollution. Moreover, a smaller C/N ratio led to the release of more effective-nitrogen.

As reported, composting at C/N ratio < 15 is favorable for nitrogen absorption by plants and would accelerate plant growth [36]. In our study, after earthworm processing, the C/N ratios in the sludge declined from 33.8 to 14.5. The C/N ratio in the wormcast is within 10–25 for any group. During this process, the earthworms not only enhanced the microbial activity, but owing to their unique eco-functions, also contributed to the minimized, stabilized, harmless and recycled use of wastes.

### Growth of *E*. *fetida* after different treatments

**Fig 1:**
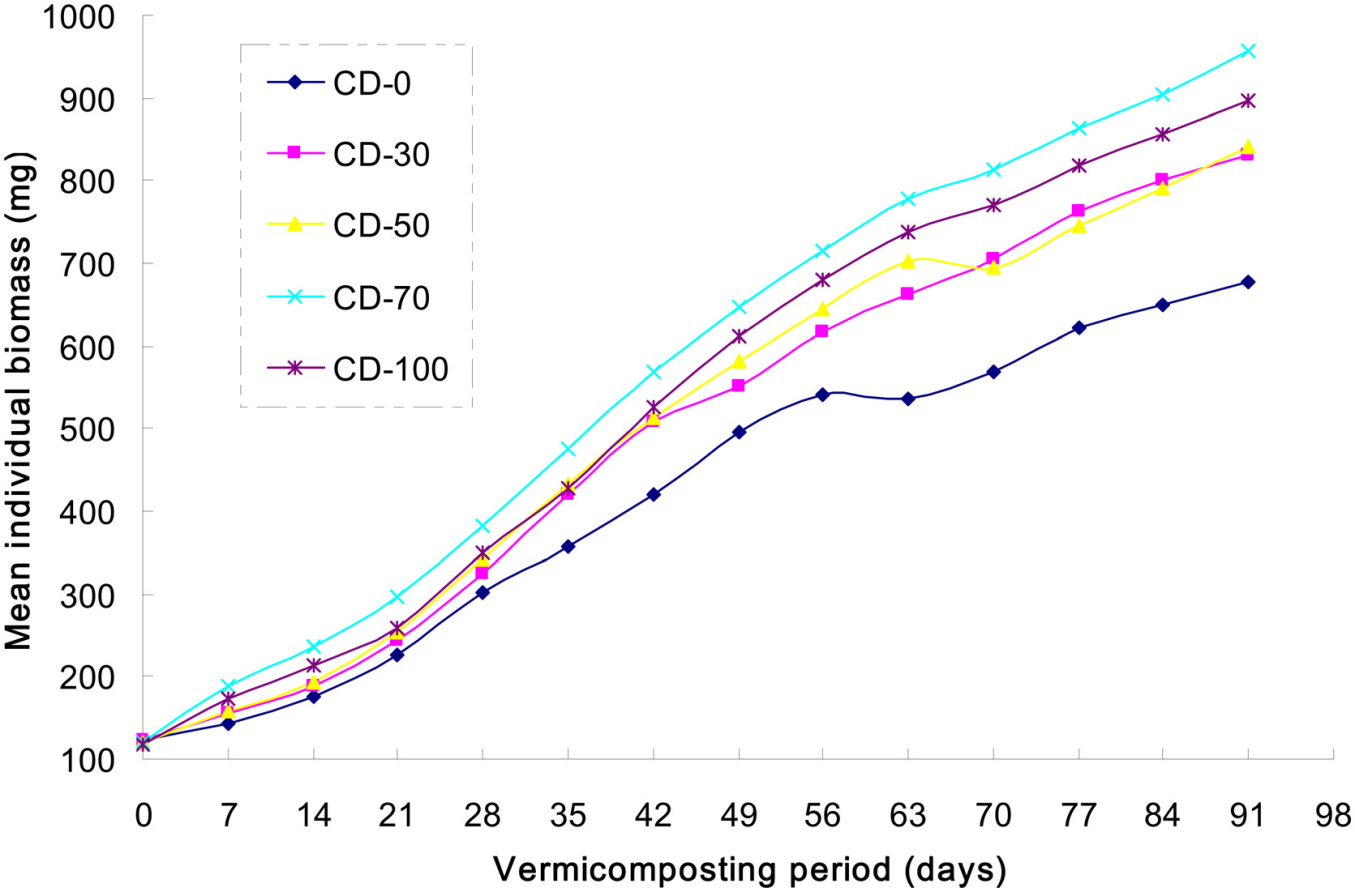
Growth curves of *Eisenia fetida* in different waste mixtures during vermicomposting.

Fig 1 shows the growth curves of *E*. *fetida* in different substrates, and Table 3 lists the growing rates. At the end of the experiments, the average biomass maximized to 910 mg after treatment CD-70, with an average growth rate of 9.20 mg·worm^-1^·day^-1^. Within the test period, the weight of earthworms did not maximize (Fig 1). The average growth rates on day 0–28, day 29–56, and day 57–91 were 9.33, 11.90, and 6.95 mg·worm^-1^·day^-1^, respectively. LSD shows significant difference in CD-70 vs. CD-0, CD-70 vs. CD-30, but not in CD-70 vs. CD-50 or CD-70 vs. CD-100 (Table 3). On day 0–28, the maximum growth rate is found after the treatment CD-70, which is significantly higher from other treatments. On day 29–56, the growth rate after treatment CD-0 is reduced, which is significantly slower from other treatments. On day 57–91, the growth rates are slower after all treatments, indicating the entrance of the breeding season decelerated the growth of *E*. *fetida*. During the test period, the growth rate after treatment CD-0 was very slow and significantly slower from other treatments, which are consistent with the growth rates at different stages. Since only sewage sludge was used in CD-0, which restricted the earthworm growth compared with other treatments added with cattle manure.

**Table 3:** The growth rate of *Eisenia fetida* among the all treatments (mg·worm^-1^·day^-1^).

Treatment	Growth rate (mg·worm^-1^·day^-1^)			
	0–28 day	29–56 day	57–91 day	0–91 day
**CD-0**	6.40±0.47^c^	8.59±0.92^b^	3.89±0.44^c^	6.11±0.77^b^
**CD-30**	7.20±1.23^bc^	10.51±1.15^a^	6.09±0.85^b^	7.79±1.17^a^
**CD-50**	7.94±0.68^b^	10.82±1.35^a^	5.64±0.62^b^	7.94±1.24^a^
**CD-70**	9.33±0.62^a^	11.90±1.69^a^	6.95±0.79^a^	9.20±1.08^a^
**CD-100**	8.21±0.72^ab^	11.84±1.08^a^	6.18±0.71^ab^	8.55±1.65^a^
***F-value***	11.60***	6.76***	16.07***	5.40**

Note: In the same column, means followed by the same superscript are not significantly different, while the means with different superscripts are significantly different (the same below).

**: 0.001<*P*<0.01,

***: *P*<0.001.

Growth rate is a good indicator that can be used to compare the growth conditions of earthworms in different wastes. Our results of growth rate are slower compared with a study on *E*. *andrei* in sewage sludge (15.63 ± 0.42, test period = 7 weeks), probably due to the difference in earthworm varieties. As reported, the weight gain rate of *E*. *fetida* depends on the population density and food type [37]. When horse manure was used as substrate and each bucket was added with 3 and 16 earthworms, the growth rates were 19 and 7 mg·worm^-1^·day^-1^, respectively [37]. With cattle manure as substrate and after 150 days, the growth rate of *E*. *fetida* was 7 mg·worm^-1^·day^-1^ [38].

### Sexual development and propagation of *E*. *fetida* after different treatments

**Fig 2:**
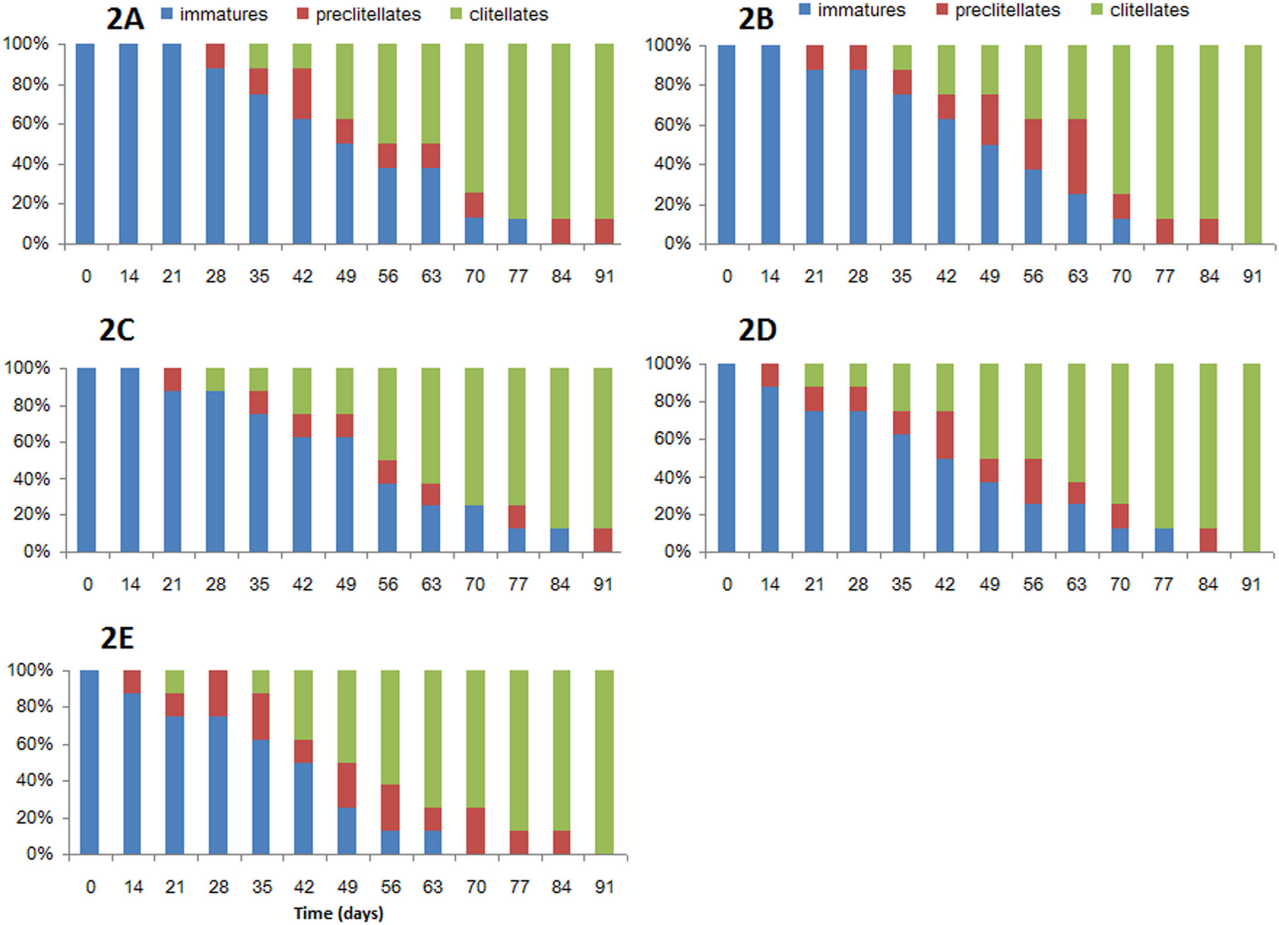
Sexual development of *Eisenia fetida* in the different diets.

Fig 2 shows the sexual development of *E*. *fetida* after treatment CD-0 (2A), CD-30 (2B), CD-50 (2C), CD-70 (2D) and CD-100 (2C). Depending on the clitellum development state, the earthworms were divided into 3 types: (l) immature worms (no evidence of external sexual characteristics); (2) preclitellates (with tubercula pubertatis- thickened structure on abdominal surfaces); (3) clitellates (fully-developed clitellum). The histograms represent the proportions of immatures, preclitellates and clitellates at different test periods. During the test periods, all earthworms developed the reproductive ring, which appeared at day 28, 21, 21, 14, and 14 after treatment CD-0, CD-30, CD-50, CD-70 and CD-100, respectively. The cocoon production started at day 35 after CD-0; the reproductive ring appeared day 35, 28, 28 and 28 after treatment CD-30, CD-50, CD-70 and CD-100, respectively. As reported, the binary combination of buffalo dung with wheat straw significantly initiated the clitellum development at 14±2.4 days, while it initiated the cocoon production at significantly earliest 30±3.4 days, and the reproduction rate was significantly highest (0.16±0.016 cocoons/worm/day) [39].

Table 4 shows the propagation of *E*. *fetida* after different treatments. During the test periods, the accumulated cocoon production changes significantly among the five treatments and it is maximized to 233 after treatment CD-70.

**Table 4:** Reproduction of *Eisenia fetida* in different waste mixtures (mean ± S.D, n = 6).

Treatment	Cumulative cocoon production during trails	Cocoon production rate (Cocoon·worm^-1^)	No. of cocoon production (Cocoon·worm^-1^·day^-1^))
**CD-0**	127±7.11^d^	15.88±0.89^d^	0.17±0.01^d^
**CD-30**	153±8.18^c^	19.13±1.02^c^	0.21±0.01^c^
**CD-50**	211±14.35^b^	26.38±1.79^b^	0.29±0.02^b^
**CD-70**	233±10.5^a^	29.16±1.31^a^	0.32±0.01^a^
**CD-100**	215±17.66^b^	26.88±2.21^b^	0.30±0.02^b^
***F*-value**	92.58***		

Note: In the same column, means followed by the same superscript are not significantly different, while the means with different superscripts are significantly different.

***: *P*<0.001.

The daily cocoon production rate is defined as the average cocoon number per day per earthworm, and it changes significantly with time. The maximum value is 0.32, and the average value is within 0.17–0.32 (cocoons·worm^-1^·day^-1^). These results are smaller than another study, probably due to the differences in food sources. For instance, the daily cocoon production rate during the 60-day period was 0.41 ± 0.05 (cattle manure as medium) with *P*. *excavatus* and 0.46 ± 0.03 with *P*. *sansibaricus* [4]. In our study, the mixtures of sewage sludge and cattle manure at certain ratios are good materials for earthworm composting, and especially the egg laying rate is maximized after treatment CD-70.

As reported, the daily cocoon production rate per earthworm is 0.15 after treatment by *E*. *fetida* at 30°C for 210 days [21] and is 0.33 after treatment at 25°C [38].

Domínguez et al. prepared mixtures from urban sludge with waste paper, waste paper box, weeds, pine needles, sawdust or food waste (dry weight 1: 1) and studied their effects on the growth and progenition of earthworms[40–41]. They found the propagation rates were relatively fast after feeding on sludge mixtures added with waste paper or paper box compared with the control (only sludge) and were 2.82±0.39, 3.19±0.30 and 0.05±0.01 cocoons earthworm^-1^ week^-1^ cocoon/week, respectively. Moreover, because of differences in the mixture properties, the earthworms utilized the energy into either growth or propagation, but not both [40–41]. The incubation from cocoons to hatchlings is affected by different factors, especially temperature. As reported, the hatching success rate was 72% at 25°C and 48% at 25–37°C, and generally, the hatching cocoons produced only one earthworm per cocoon [38]. Moreover, cow manure contributed to larger hatchling production compared with dry leaves [42].

### Heavy metal contents in mixtures after different treatments

Sewage sludge and cattle manure both contain certain amounts of harmful heavy metals, such as Sb, Pb, Zn, Cd, Cu, and Ni. The heavy metal concentrations of sewage sludge and cattle manure in different treatments are listed in Table 5. ANOVA shows that the Sb concentrations after treatment CD-100 are significantly different from those after other treatments, but are not significantly different after treatments CD-0, CD-30, CD-50 and CD-70. The possible reason is that the sewage sludge contains more Sb than cattle manure. The Pb concentrations are significantly different between treatments CD-70 and CD-100, but the Zn and Pb contents are both similar. The Cd concentrations are significantly different among treatments CD-50, CD-70 and CD-100.

**Table 5:** Contents of heavy metals of sewage sludge and cattle dung manure in the different treatments (mg/kg).

Treatment	Antimony	Lead	Zinc	Cadmium
**CD-0**	3.36±0.09^a^	43.38±1.55^ab^	282.83±8.16^a^	5.65±0.11^a^
**CD-30**	3.29±0.14^a^	44.65±1.31^a^	278.93±4.88^ab^	5.66±0.14^a^
**CD-50**	3.28±0.12^a^	43.42±1.42^ab^	278.31±6.54^ab^	5.49±0.1^b^
**CD-70**	3.24±0.13^a^	42.18±1.08^b^	275.14±5.92^b^	5.45±0.11^bc^
**CD-100**	3.22±0.88^b^	43.19±1.03^ab^	275.38±5.24^b^	5.34±0.08^c^
***F*-value**	1.27	2.78*	1.50	9.48***

Note: In the same column, means followed by the same superscript are not significantly different, while the means with different superscripts are significantly different.

*: 0.01<*P*<0.05,

***: *P*<0.001.

Earthworms which accumulate abundant metals in their tissues can serve as a useful biological indicator of contamination, because the concentrations of some contaminants in vivo are consistently related [43–44].

As reported, heavy metal concentrations (Cd, Pb and Zn) decreased in the vermicompost than in the initial sludge due to the adsorption by *E*. *fetida*, and those in earthworm tissues increased. The mean adsorptions at the end of the experiments were 155.15±7.49 (Zn), 71±5.822 (Pb) and 58.69±1.67 (Ni) mg/kg [45]. As reported, in earthworm composting experiments, the Cd, Zn and Pb contents are 7.64±0.35, 109.85±7.18 and 51.33±1.97 mg/kg, respectively, and the bio-enrichment factors of Cd, Zn and Pb are 2.973, 1.316, and 0.443, respectively [45]. These results indicate *E*. *fetida* can modestly enrich Cd and Zn and helps to remove some heavy metals from sludge. Sb concentrations in sewage sludge and cattle manure are relatively low. The bio-enrichment factor of Sb is 0.64, indicating *E*. *fetida* has low enrichment ability over Sb. The 14-day acute toxicity tests of *E*. *fetida* show that the median lethal concentration of Cd is 1118 mg/kg. In our study, the Cd concentration in *E*. *fetida* is 5.34–5.66 mg/kg, but Cd has little effect on *E*. *fetida*. Thus, *E*. *fetida* can be used to dispose compounds from sewage sludge and cattle manure.

## Conclusions

The physiochemical properties of materials depend on food structures, which affect not only the number, but also growth and propagation, of *E*. *fetida*. After earthworm composting, the physiochemical properties of materials are largely changed. Among all treatments, the TOC, TP, pH, EC and C/N ratio all decreased to different extents, but TKNs all increased. At three growth stages (day 0–28, day 29–56 and day 57–91), the average growth rates after all treatments changed in the trend of "rapid—rapidest—slow growth". After treatment with pure sludge (CD-0), the growth of *E*. *fetida* was restricted to some extent compared with other treatments added with cattle manure. The reproductive rings after different treatments appeared at different time points, and the starting time of cocoon production was also varying, indicating food structures largely affected the cocoon production time of *E*. *fetida*. Different treatments affected the heavy metal contents differently, but slightly affected the growth and reproduction of *E*. *fetida*. *E*.*fetida* could enrich Cd largely, but enrich Sb slightly. Earthworm composting facilitates the removal of some heavy metals.
